# Higher Number of Night Shifts Associates with Good Perception of Work Capacity and Optimal Lung Function but Correlates with Increased Oxidative Damage and Telomere Attrition

**DOI:** 10.1155/2019/8327629

**Published:** 2019-04-11

**Authors:** Sofia Pavanello, Mariarita Stendardo, Giuseppe Mastrangelo, Valeria Casillo, Marco Nardini, Antonio Mutti, Manuela Campisi, Roberta Andreoli, Piera Boschetto

**Affiliations:** ^1^Occupational Medicine, Department of Cardiac, Thoracic, and Vascular Sciences and Public Health, University of Padova, Padova, Italy; ^2^Department of Medical Sciences, University of Ferrara, Ferrara, Italy; ^3^Department of Prevention and Protection, University-Hospital and Public Health Service of Ferrara, Ferrara, Italy; ^4^Department of Medicine and Surgery, University of Parma, Parma, Italy; ^5^CERT Centre for Excellence in Toxicology Research, University of Parma, Parma, Italy

## Abstract

Sleep deprivation and the consequent circadian clock disruption has become an emergent health question being associated with premature aging and earlier chronic diseases onset. Night-shift work leads to circadian clock misalignment, which is linked to several age-related diseases. However, mechanisms of this association are not well understood. Aim of this study is to explore in night-shift workers early indicators of oxidative stress response and biological aging [oxidized/methylated DNA bases and leukocytes telomere length (LTL)] and late indicators of functional aging [lung function measurements (FEV1 and FVC)] in relation to personal evaluation of work capacity, measured by work ability index (WAI). One hundred fifty-five hospital workers were studied within the framework of a cross-sectional study. We collected physiological, pathological, and occupational history including pack-years, alcohol consumption, physical activity, and night shifts, together with blood and urine samples. Relationships were appraised by univariate and multivariate ordered-logistic regression models. We found that workers with good and excellent WAI present higher FEV1 (p< 0.01) and number of night-work shifts (p<0.05), but they reveal higher urinary levels of 8-oxoGua (p<0.01) and shorter LTL (p<0.05). We confirmed that higher work ability was prevalent among chronological younger workers (p<0.05), who have also a significant reduced number of diseases, particularly chronic (p<0.01) and musculoskeletal diseases (p<0.01). The new findings which stem from our work are that subjects with the highest work ability perception may have more demanding and burdensome tasks; they in fact present the highest number of night-shift work and produce unbalanced oxidative stress response that might induce premature aging.

## 1. Introduction

The growing aging of the population is an emerging social and public health problem, even in the workplace. The extension of life expectancy has entailed a longer permanence in the working world so much that in most industrialized countries the workforce is grappling with the “Silver Tsunami” [1]. Prolonged working lives underscore importance for public health in recognizing a close connection of work ability with aging and health [2]. By age of 50 years, half of the population have at least one chronic disease and are three times more likely to report that they are unable to work due to health problems [3].

Sleep deprivation and the consequent circadian clock disruption has become an emerging health issue being associated with premature aging and early onset of chronic conditions including obesity, cardiovascular diseases (CVD), metabolic diseases (MetS), and cancer [4]. Night-shift work leads to circadian clock misalignment, which is linked to several age-related diseases [5, 6]. However, the mechanisms of this association are not well understood. With this respect, oxidative stress has been showed to be associated with night-shift work [7, 8] and therefore to be a plausible pathophysiological mechanism through which night-shift work may influence the observed risk of age-related-diseases [9].

Aging is characterized by a progressive loss of biological/physiological integrity, leading to compromised function that increases vulnerability to illness and death [10]. However, aging cannot simply refer to the effect of the chronological time course, given that it is an individual, complex, biological process modulated by internal and external factors [10]. Thus, age, when measured chronologically, may not be a reliable indicator of the real psychophysical decline over years [11]. Biological and functional aging is assumed to reflect the ongoing change within a person, the intrinsic body's degeneration, and its capacity of response to different stressors (i.e., genetic factors and exposure to environmental and occupational agents) [11, 12]. In the complex framework of biological aging a variety of molecular, biochemical and metabolic changes occur at the cellular level. Oxidative damage to biomolecules (nucleic acids, lipids, and proteins) and altered methylation pattern assessed through oxidized and methylated nucleic acids in urine and erosion of telomere length, measured in peripheral blood leukocytes (LTL), are considered early hallmarks of biological aging, which primary cause damage to cellular functions [10]. Boosted inflammatory responses [13] and exposure to environmental [14] and occupational stimuli [15], including perceived stress at work [16, 17], favoring the oxidative stress, can speed up the physiological telomere attrition [18] and increase the risk of age-linked disorders, i.e., chronic degenerative disease, including cancer [19].

Lung function assessment is a useful tool in functional aging and health assessment, representing a later clinical phenotype as a consequence of aging cellular damage accumulation [20, 21]. Findings demonstrate a consistent pattern of decline of Forced Expiratory Volume in one second (FEV1) and Forced Vital Capacity (FVC) with the advancing years. Furthermore, spirometry measures predict the development of CVD, cognitive decline, and mortality in the general population [22].

Work capacity, as measured by work ability index (WAI), refers to an individual perception of the balance between work demands and the ability to cope with them [23]. Hence, work capacity is a dynamic process that results from the interaction among psychosocial and physical work-related factors, mental and physical capabilities, and health conditions [24, 25]. Work capacity changes greatly throughout working life: generally, there is a decreasing trend over the years and chronic diseases, whose number typically increases with age, are associated with a poor work ability [26, 27]. However, inconsistent relations between age and work capacity have also been observed. It is likely that significant heterogeneity exists between individuals in their trajectories of perceived work capacity with age. These findings highlight the needs to better understand nuances of the relationship between work ability and age [23]. Biological and functional indicators of aging can provide a new insight of the heterogeneity of age-related psychophysical changes throughout working life [28].

The aim of this study was to explore the relationships among WAI, i.e., a subjective evaluation of personal resources with respect to work demand, and night-shift work, with early indicators of biological aging, such as markers of oxidative and altered methylation damage to nucleic acids and LTL, and late indicators of functional aging, like FEV1 measurements, in a group of hospital workers. Given the multifactorial structure of work ability, work schedule, health status, and behavioral characteristics of each participant were considered.

## 2. Materials and Methods

### 2.1. Subjects and Study Design

The present study included nurses, aged between 18 and 65 years, recruited from an earlier cross-sectional study conducted from March 2015 through July 2015, fully described in a previous publication [29]. Briefly, participants were interviewed to collect demographic data, lifetime smoking history, alcohol consumption, dietary habits, and physical activity in leisure time [International Physical Activity Questionnaire (IPAQ score)]. Occupational history was also collected including data on number of night shifts in a month, work injury, and work ability by WAI were collected as previously described [29]. Work shifts included day shifts (6 a.m.–2 p.m.), afternoon shift (2 p.m.–8 p.m.), and night shifts (8 p.m.–6 p.m.).

Diseases, registered by medical history, were classified in 9 main groups: CVD, arterial hypertension, musculoskeletal disease, spinal disk hernia, gastrointestinal disease, endocrine disease, diabetes, respiratory disease, and tumors. The Charlson comorbidity index (CI), a test predicting the risk of death by measuring the burden of comorbidities, was also calculated [30]. We did not include diabetes, tumors, respiratory diseases, and other inflammatory disorders in the Charlson assessment.

Study subjects underwent also a complete physical examination as previously described [29], assessment of pulmonary function. We also collected (1) a fasting blood sample for basic biochemistry, C-reactive protein (CRP), and telomere length determination and (2) a urine sample to determine both nucleic acid oxidation and methylation biomarkers.

The study was authorized by the local Ethics Committee and conducted in accordance with the ethical standards of the Declaration of Helsinki and its later amendments. A written informed consent was obtained from all participants.

### 2.2. Work Ability Assessment

To evaluate work ability, the Work Ability Index (WAI) was used as previously described [31]. Briefly, the WAI consists of 7 domains. The total WAI score is the sum score of the 7 domains and ranges from 7 to 49. The WAI is classified into “poor” (score 7–27), “moderate” (score 28–36), “good” (score 37–43), and “excellent” (score 44–49) work ability. Among study participants there were only 5 subjects with "poor" work ability; therefore, for our analysis, poor and moderate work ability were aggregated into one category. Thus, participants were grouped into 3 classes: (1) poor-moderate (n=57 subjects), (2) good (n=85 subjects), and (3) excellent work ability (n=13 subjects).

### 2.3. Spirometry

Forced expiratory volume in one second (FEV1), forced vital capacity (FVC), and the FEV1/FVC ratio were measured using a spirometer (Biomedin, Padova, Italy). The best of three values of FEV1 and FVC were expressed in litres and as a percentage of the predicted normal value. All measurements were obtained and interpreted in accordance with the recommendations of the American Thoracic Society/European Respiratory Society (ATS/ERS) [32].

### 2.4. Laboratory Tests

#### 2.4.1. Basic Biochemistry

Data on biochemistry included number of blood red cells, platelets, lymphocytes, monocytes, neutrophils, basophils, eosinophils, and white cells, hemoglobin, glycated hemoglobin, blood glucose, triglycerides, cholesterol, low density lipoprotein, high density lipoprotein, and c-reactive protein.

#### 2.4.2. Lymphocytes Telomere Length (LTL) Analysis

LTL was determined after DNA extraction as previously described [29]. LTL was appraised by the real-time quantitative polymerase chain reaction (PCR) method established by Cawthon [33] as we previously developed [14, 15, 29]. In brief, this method measures the relative TL by estimating in genomic DNA the proportion of telomere repeat copy number (T) in relation to a single copy gene (S) (T:S fraction) as previously described [14, 15]. The single copy gene employed in this investigation was, as previously described, the human *β*-globin (hbg). The PCR runs were conducted on a SteponePlus Real Time PCR System (Thermo Fisher Scientific, Italy). The reproducibility of LTL measurements was verified by replicating 15 samples 3 times on 3 different days. The within-sample coefficient of variation (CV) for the mean T/S ratio on 3 successive days was 8.5%, which was comparable to the CV described for the earliest protocol as previously described [29, 33].

#### 2.4.3. Oxidized and Methylated Nucleic Acids Analysis

Biomarkers of nucleic acids oxidation, as 8-oxo-7,8-dihydro 2'deoxyguanosine (8-oxodGuo), 8-oxo-7,8-dihydro-guanosine (8-oxoGuo), and 8-oxo-7,8-dihydroguanine (8-oxoGua), and methylation, such as 5-methyl-cytosine (5-MeCyto), 5-hydroxymethyl-cytosine (5-OHMeCyto), 5-methylcytidine (5-MeCyt), 5-methyl-2'deoxycytidine (5-MedCyt), 5-hydroxymethyl-2'deoxycytidine (5-OHMedCyt), 1-methyl-guanine (1-MeGua), 7-methyl-guanine (7-MeGua), and 7-methyl-guanosine (7-MeGuo), were determined by isotopic dilution liquid chromatography tandem mass spectrometry (LC-MS/MS) using a API 4000 triple quadrupole mass spectrometer (SCIEX, Framingham, MA USA) equipped with a TurboIonSpray interface for pneumatically assisted electrospray according to the method by Andreoli et al. [34], with some modifications to determine urinary methylated biomarkers in the same chromatographic run. Briefly, after centrifugation urinary samples (50 *μ*L) were diluted with 150 *μ*L of internal standards (including [^13^C_1_,  ^15^N_2_] 8-oxo-7,8-dihydroguanine, [^15^N_5_] 8-oxo-7,8-dihydro-guanosine, [^15^N_5_] 8-oxo-7,8-dihydro 2'deoxyguanosine, and 5-hydroxymethyl-2'deoxycytidine-d_3_) aqueous mixture and injected (5 *μ*l). An Atlantis®dC18 column (100 x 2.0 mm i.d., 3 *μ*m; Waters, Milford, MA) was used to perform the separation of urinary analytes, obtained with variable proportions of 10 mmol/l aqueous formic acid (pH 3.75) and methanol at a flow-rate of 0.2 mL/min, with a make-up flow of methanol (0.07 mL/min) after column separation. Positive ion mode was applied to obtain the ionization of all biomarkers and the acquisition was performed in selected reaction monitoring mode. For quantitative analysis, working calibrations were obtained by spiking pooled urines with standard solutions at different concentration levels, specific for each analyte. Calibration curves were constructed by linear regression analysis of the analyte-to-IS area ratio versus the known concentration of analytes injected (r^2^>0.998). The limits of quantification (LOQs) were between 0.75 pmol/mL for 8-oxodGuo and 8-oxoGuo, and 10 pmol/mL for 5-OHMeCyto, respectively. The (%CV) ranged between 2.0% and 6.8% for all analytes and for all intra- and interday determinations. In all analyzed samples, the urinary levels of biomarkers of nucleic acids oxidation and methylation were higher than LOQs. In the past, the laboratory that performed the urinary biomarkers determinations was involved in an interlaboratory project which included quality control to assess urinary concentration of 8-oxodGuo, organized by ESCULA [35]. Concentrations of urinary metabolites were expressed as a function of creatinine concentration (pmol/*μ*molcreat), measured by the method of Jaffe [36].

We adopted the exclusion criteria of the American Conference of Governmental Industrial Hygienists. Therefore, urine samples with creatinine concentrations lower than 2.65 *μ*mol/mL or with creatinine concentration higher than 26.5 *μ*mol/mL were excluded (American Conference of Governmental Industrial Hygienists) [37].

### 2.5. Statistical Analysis

At univariable analysis, the diversities among the groups of WAI were appraised with Kruskal–Wallis one-way analysis of variance (interval variables) or Chi-square test (frequency variables). A p-value of 0.05 was the threshold for statistical significance.

At multivariable analysis, the outcome variable “WAI” with three levels (0=“poor-medium”; 1=“good”; 2=“very good” subset) was the dependent variable in a model of ordered-logistic regression (OLR), assuming that levels of WAI had a natural sorting (from low to high) even though the gaps between adjacent levels were unidentified.

Since it estimates only one equation for all levels of the dependent variable, OLR model only applies to data that meet the proportional odds assumption. To test proportionality assumption, we used the Brant test that verifies the parallel regression hypothesis. The statistics of the Brant test (Chi-square = 8.079; degrees of freedom = 5; p = 0.152) confirmed the supposition of parallel regression.

The predictive variables were chosen among the many independent variables with a stepwise program of automatic selection [forward (p=0.05) and backward (p=0.06) selection].

OLR program estimates a regression coefficient (Coef.) according the “null” hypothesis" that the value of a predictor is zero. The explanation of the coefficient is that, for the increase in one unit of the predictor, the level of the response variable changes according to the value of the respective regression coefficient expressed in log-odds scale, while the other variables in the model are kept constant. A negative or positive sign of the coefficient indicate a negative or positive association between WAI and predictive variables. For each regression coefficient, the program provides the 95% confidence interval (95%CI)—which expresses the 95% confidence that the “true” population regression coefficient is placed between the lower and upper limit of the interval—and a two-tails probability of error (p).

## 3. Results

Table 1(a) shows interval variables (mean ± SD) according to WAI score ranked in three groups of subjects [n=57 poor (from 7 to 27) and moderate (from 28 to 36), n=85 good (from 37 to 43), and n=13 excellent (from 44 to 49)]. Kruskal–Wallis analysis of the variance indicates that workers with good and excellent work ability are younger (p = 0.0413) and with higher FEV1 (p = 0.0028), but they present higher urinary levels of 8-oxoGua (p = 0.0368).

Table 1(b) shows the number and percentage of frequency variables in the same three groups of WAI. The Chi-square test showed that workers with chronic (p<0.001), musculoskeletal (p<0.001), and spinal disk hernia diseases (p<0.0001) and, less distinctly, those with endocrine (p = 0.020) and gastrointestinal (p = 0.033) diseases present poor-moderate WAI, whereas workers with excellent work ability were prevalently male (p = 0.019) and present higher number of night-work shifts (53.9%) compared to those with poor-moderate WAI (28.3%) (p=0.043).

All the other characteristics were instead equally distributed among the three groups.

Table 2 shows the results of the ordered-logistic regression model with the response variable WAI and stepwise selection of predictors. The first column displays the predictors entered the model: chronic and musculoskeletal disease (dichotomous variables coded 0/1 for absence/presence of disease), FEV1, urinary 8-oxoGua, and telomere length (all continuous variables). The second column shows the ordinal regression coefficients expressed as log-odds (logit model), the 95% confidence interval, and the p-values. The results show that higher work ability is positively related to lung function assessed by FEV1 (p=0.001), although it was negatively associated with higher levels of urinary 8-oxoGua (p=0.005) and has shorter LTL (p=0.049). In addition, work ability is negatively related to chronic (p≤0.001) and musculoskeletal diseases (p=0.003), such as spinal disk hernia, endocrine, and gastrointestinal disease.

## 4. Discussion

This study suggests important implications regarding night-shift work; work ability, measured by WAI, a subjective evaluation of personal resources with respect to work demand; oxidative stress response, detected by oxidized and methylated bases excreted in free modified forms in urine; biological aging, assessed by LTL analysis; and functional aging, assessed by lung function measurements.

First, workers with good and excellent work ability exhibit a higher number of night-work shifts and in the meantime present higher levels of urinary 8-oxoGua, the most important biomarker of oxidative damage to DNA and specific of the activation of hOGG1 enzyme involved in the base DNA excision repair mechanism; second, workers, with the upper levels of work ability and higher oxidatively DNA damaged biomarker, have also shorter LTL; third, good and excellent work ability is positively related to lung function assessed by FEV1. Also, we confirmed that good/excellent work ability was prevalent among chronological younger workers who have also a significant reduced number of diseases, particularly chronic and musculoskeletal diseases, such as spinal disk hernia, endocrine, and gastrointestinal disease.

### 4.1. Night-Shift Work, Work Ability, and 8-oxoGua

Among the novel findings of this research is that elevated work ability associates with number of night-work shift and urinary concentrations of 8-oxoGua. 8-oxoGua is by far the most concentrated DNA oxidative product excreted in a free form in the urine [38–40], and it is the product of the activation of the enzyme hOGG1 glycosylases, known as a specific mechanism to remove this lesion from cellular DNA [41, 42]. Of all the modified bases, the process which repairs 8-oxoGua is perhaps the best understood. The excision product can be transported across the cell membranes and then excreted into cerebrospinal fluid, plasma, and urine without any further metabolism [43]. 8-oxoGua in urine is used to assess the oxidative stress effect on DNA and the efficiency of hOGG1 repair system [44]. The oxidatively damaged DNA reflects a state of cellular imbalance caused by ROS, in which ROS production exceeds antioxidant mechanisms that counterbalance it. Most of previous studies have stated a rise in oxidatively damaged DNA with age [45–47], even in aged mice, rats, and monkeys [48–50], while a few found no age-related difference [51]. Oxidatively damaged DNA has been associated with a number of age-related pathologies, including cancer, neurodegenerative, and cardiovascular diseases [52]. Our study would suggest that sources of oxidative stress such as night turn shift, other than chronological age and chronic age-related diseases (in fact our workers with higher oxidative damage were younger and healthier), could contribute to increase the biomarkers of oxidative DNA damage (8-oxoGua and 8-oxodGuo) but not 8-oxoGuo that are instead related to RNA breakdown [44]. In effect, the workers that performed the highest number of night-work shift have a significantly higher urinary levels of 8-oxoGua, product of the activation of a specific DNA damage, compared to workers with lower number of night-work shift. Even if not statistically different, a similar trend was observed for 8-oxodGuo, the other urinary biomarker of DNA damage and repair. Urinary concentration of biomarker of oxidative damage to RNA, 8-oxoGuo, seems to be not affected by number of night-work shift, maybe because it is related to the RNA breakdown and not by specific repaired mechanisms [44]. In agreement with our results two previous studies reported significantly greater urinary oxidative damage in night, than day-shift workers [53, 54], while a third study did not [55]. In the whole, these results can be explained by the fact that sleep disruption, which is well recognized among night-shift workers [56], has also been linked to increased levels of ROS [7, 8]. Since melatonin is both an efficient scavenger of ROS and an inducer of antioxidative enzymes [57, 58], its disruption during shift work [59, 60] might have contributed to raise the level of ROS and consequently the urinary oxidative damage.

It might be added that psychological stress has also been related to higher levels of oxidatively damaged DNA and of lipids too [17, 61–64]. Oxidative stress is also elevated in depressive disorder; a systematic review and meta-analysis found increased oxidative stress markers in major depressive disorder, with 8-oxodGuo being the most prominent biomarker [65]. Based on these premises, our results could suggest that our workers might be under psychological stress. Those subjects with the highest working skills have perhaps more demanding and burdensome tasks. Finally, WAI, an indicator of the balance between personal resources, especially in relation to chronic disorders, and work demand, would not be able to detect the psychological stress in these workers.

### 4.2. Night-Shift Work, Work Ability, and Telomere Length

Another novel finding is that the upper grades of WAI are related to shorter LTL. Telomeres are DNA repeat sequences (TTAGGG)n that, together with combined proteins, create a sheltering complex that guards chromosomal ends and cares for their integrity [66]. Due to restriction of DNA polymerase (telomerase), genetic stability is progressively deprived as telomeres shorten with each round of cell division [66, 67]. Furthermore, telomere attrition can even be further quickened by external stressors. Telomeres, in fact, as triple-guanine-containing sequences, are highly susceptible to genotoxic damage including oxidative stress [68]. The resulting damaged telomeric nucleobases by double-strand breaks and/or interference with replication fork induce a reduction in telomere length [18]. Therefore the higher level of oxidative damage in workers with good/excellent work ability could be the biological explanation of telomere erosion we found in these workers. The relationship between DNA damage induced by oxidative stress and LTL has been extensively described in vitro [69]. To our knowledge this is the first study that report in humans higher oxidatively damaged DNA and LTL attrition in relation to work ability.

It might be added that higher oxidatively damaged DNA related to shorter LTL has been even associated with psychosocial stress [62]. The mechanism seems to be mediated by stress-related hormones (e.g., cortisol) [70, 71]. Experimentally, high amounts of cortisol (a potent stress hormone) have been shown to reduce telomerase activity in leukocytes with shorter LTL [72]. A number of cross-sectional studies in humans have associated LTL erosion with a high degree of psychosocial stress [62, 73, 74] and related biomarkers [62, 74]. In our study, workers with higher levels of urinary 8-oxoGua and shorter LTL present however the highest worker ability. These workers with the highest working skills have more demanding and burdensome tasks and in fact present the highest number of night-shift works that could generate even psychological stress.

### 4.3. Work Ability and Lung Function

FEV1 positively correlates with WAI indicating another important determinant for evaluating work capacity. Lung function assessment is a useful tool in functional aging and health assessment, representing a later phenotypical/clinical consequence of aging cellular damage accumulation [20, 21]. In the tricky context of aging mechanisms, physiological function, such as lung function evaluated by FEV1, can be considered a late event arising when the accumulated cellular damage cannot be counteracted by tissue homeostatic mechanisms. The fact that FEV1 is higher in workers with good/excellent WAI category suggests that these younger workers do not still present the permanent phenotypic features of this later physiological decline.

## 5. Conclusion

In conclusion, there are new several relevant findings stemming from our study. A good/excellent perception of work ability positively correlates with FEV1 indicating, for the first time, another important determinant of work capacity evaluation. On the other hand, workers with good/excellent work capacity, presenting higher levels of 8-oxoGua, urinary product of oxidatively damaged DNA repair, reveal higher LTL erosion. As these workers perform the highest number of night shifts, it could be hypothesized that an unbalanced oxidative stress response, likely generated by sleep disruption, and perhaps by psychological stress, our workers have more demanding and burdensome tasks, would make workers more susceptible to premature aging, which we were able to detect at earlier stage before it causes an irreversible decline of functional aging. This also suggests that the WAI represents a reliable indicator of work ability in relation to clinical physiological decline, but does not reflect the accumulated burden of endogenous molecular damage that undermines the health of a worker. Accordingly, we may assert that the WAI presents a limited power in assessing the real biological condition/status of people in employment. Therefore, a complete and predictive evaluation of work capacity would require that WAI is combined with molecular indicators of biological aging.

## Figures and Tables

**Table:** (a) Interval variables (mean ± SD) by level of work ability, according to WAI scores: 7-36 (poor-moderate), 37-43 (good), and 44-49 (excellent) in the study population.

Variables	Level of work ability		
	Poor-moderate n=57	Good n=85	Excellent n=13
Age (years) *∗*	48.7±5.7	47.9±6.2	44.7±4.9
Length of employment (years)	25.0±6.1	24.5±7.9	22.0±5.5
Length in the current job (years)	14.6±8.9	18.0±9.7	15.9±7.6
Education (years)	15.0±1.5	15.0±1.6	14.9±1.7
Body mass index (kg/m^2^)	27.2±6.1	25.4±4.6	24.0±3.6
Waist (cm)	99.2±13.7	96.0±12.0	93.8±7.7
Systolic pressure (mm Hg)	119.9±17.2	115.2±12.8	111.5±10.7
Diastolic pressure (mm Hg)	75.4±10.7	75.0±9.2	71.4±10.0
Mother age (years)	27.4±5.5	28.2±7.1	27.2±6.3
Father age (years)	30.1±5.2	31.8±7.0	29.9±6.1
Pack years [(cigarettes/20) x years]	7.7±10.1	6.9±10.4	10.2±10.5
Drinking (age at start, years)	13.0±11.3	13.5±11.8	17.2±10.7
Alcohol (daily intake last year)	0.2±0.3	0.2±0.4	0.3±0.8
Sport (IPAQ score)	3.2±3.4	3.4±2.3	2.8±2.1
Leukocytes (10^3^/ml)	6.8±1.6	6.9±1.9	7.5±2.2
Blood red cells (10^3^/ml)	4.6±0.6	4.7±0.4	4.6±0.5
Hemoglobin (g/dl)	12.8±1.4	13.3±1.4	13.7±1.6
Platelet count (10^3^/ml)	282.4±71.6	279.6±63.8	247.7±51.9
Neutrophils (10^3^/ml)	3.5±1.1	3.6±1.3	3.9±1.4
Lymphocytes (10^3^/ml)	2.5±0.7	2.5±0.8	2.8±1.2
Monocytes (10^3^/ml)	0.6±0.2	0.6±0.2	0.6±0.2
Eosinophils (10^3^/ml)	0.2±0.1	0.2±0.1	0.2±0.1
Basophils (10^3^/ml)	0.03±0.02	0.03±0.02	0.03±0.01
Glycaemia (mg/dl)	83.3±15.7	83.7±12.5	80.4±12.6
Cholesterol (mg/dl)	201.6±42.8	210.1±37.1	189.5±41.7
Triglycerides (mg/dl)	96.8±44.7	97.6±52.4	75.5±32.5
Low-density lipoprotein (mg/dl)	119.6±35.9	120.2±34.9	107.6±36.3
High-density lipoprotein (mg/dl)	65.4±19.2	69.9±16.6	66.8±15.1
C-reactive protein (mg/ml)	0.4±0.4	0.4±0.2	0.4±0.2
Glycated hemoglobin (mmol/mol)	36.4±7.0	36.5±7.4	32.0±5.8
Leukocyte telomere length (T/S)	1.1±0.2	1.1±0.2	1.0±0.2
FEV_1_ (liters) *∗∗*	2.8±0.5	2.9±0.6	3.5±0.7
FEV_1_ (% predicted)	105.7±15.8	108.4±11.9	113.6±19.9
Creatinine urine (*μ*mol/mL)	9.4±4.7	10.0±5.1	10.6±6.2
8-oxoGuo (pmol/*μ*mol_creat_)	17.4±8.9	17.6±7.9	17.8±9.3
8-oxoGua (pmol/*μ*mol_creat_)*∗*	73.5±47.6	104.8±77.3	84.3±60.2
8-oxodGuo (pmol/*μ*mol_creat_)	8.4±5.0	9.3±5.2	11.4±7.5
7-MeGua (nmol/*μ*mol_creat_)	29.8±9.0	30.1±11.4	30.9±10.3
1-MeGua (pmol/*μ*mol_creat_)	887.5±1231.9	820.9±847.8	677.2±343.6
7-MeGuo (pmol/*μ*mol_creat_)	206.5±143.1	187.3±135.9	155.2±81.8
5-MeCyto (pmol/*μ*mol_creat_)	368.1±297.5	399.1±267.9	442.0±389.9
5-MeCyt (pmol/*μ*mol_creat_)	19.1±16.5	26.2±38.8	24.9±26.4
5-MedCyt (pmol/*μ*mol_creat_)	12.6±13.3	14.1±15.5	25.0±20.6
5-OHMeCyto (pmol/*μ*mol_creat_)	82.1±148.5	65.7±115.3	63.9±74.1
5-OHMedCyt (pmol/*μ*mol_creat_)	10.2±5.8	10.7±7.9	13.5±9.1

p-values (Kruskal–Wallis one-way analysis of variance): *∗* <0.05; *∗∗* = 0.002.

**Table:** (b) Frequency variables (percentage) by level of work ability, according to WAI scores: 7-36 (poor-moderate), 37-43 (good), and 44-49 (excellent) in the study population. For dichotomous variable, table reports the percentage of individuals belonging to the class coded as 1.

Variables	Classes	Levels of work ability		
		Poor-moderate n=57	Good n=85	Excellent n=13
Sex *∗*	Male	5.3	9.4	30.8
Shift work	Night shift workers	38.6	48.2	61.5
Nights at work	0	61.4	51.8	38.5
	2-4	10.6	17.7	7.7
	≥5*∗*	28.1	30.6	53.9
Work injury	1 or more	29.8	32.9	15.4
Smoking	Non smokers	38.6	52.9	38.5
	Ex-smokers	29.8	20.0	23.1
	Smokers	31.6	27.1	38.5
Drink	Drinkers	61.4	63.5	76.9
Binge	None	94.7	89.4	92.3
	4	5.3	8.2	7.7
	≥5	0.0	2.4	0.0
Chronic disease *∗∗*	1 or more	98.3	75.3	15.4
Charlson index	≥1	24.5	17.6	0.0
Musculoskeletal disease *∗∗*	1 or more	79.0	55.3	15.4
Spinal disk hernia *∗∗*	1 or more	68.4	43.5	15.4
Cardiovascular disease	1 or more	24.6	17.7	0.0
Arterial hypertension	Yes	21.1	14.1	0.0
Gastrointestinal disease *∗*	1 or more	33.3	22.4	0.0
Endocrine disease *∗*	1 or more	36.8	24.7	0.0
Diabetes	Yes	5.3	4.7	0.0
Respiratory	Yes	15.8	7.1	0.0
Tumors	Yes	8.8	7.1	0.0

p-values (Chi-square test): *∗* <0.05; *∗∗* <0.001.

**Table 2:** Ordered logistic regression analysis of the association between WAI and biological and clinical variables.

Variables	Coef.	95% CI	p
Chronic disease	-2.653	-1.210; -4.095	≤0.001
Musculoskeletal disease	-1.437	-0.477; -2.397	0.003
FEV_1_ (liters)	1.242	0.520; 1.963	0.001
8-oxoGua (pmol/*μ*mol_creat_)	0.007	0.002; 0.013	0.005
Leucocyte telomere length (T/S)	-1.638	- 0.008; -3.267	0.049

Coef. = coefficient of ordered regression expressed as log odds; 95% CI = 95% confidence interval.

## Data Availability

The data on demographics (age and gender) and other pieces of personal information (mother/father age at birth and years of education), physiological, pathological, and occupational anamnesis including pack-years, alcohol consumption, physical activity, and frequency of night shifts, used to support the findings of this study are available from the corresponding author upon request.
